# Baseline predictors of treatment gains in peak propulsive force in individuals poststroke

**DOI:** 10.1186/s12984-016-0113-1

**Published:** 2016-01-15

**Authors:** HaoYuan Hsiao, Jill S. Higginson, Stuart A. Binder-Macleod

**Affiliations:** Biomechanics and Movement Science Program, University of Delaware, Newark, DE 19716 USA; Department of Mechanical Engineering, University of Delaware, Newark, DE 19716 USA; Department of Physical Therapy, University of Delaware, Newark, DE 19716 USA; 540 S. College Avenue, Suite 201F, Newark, DE 19716 USA

**Keywords:** Stroke, Gait, Propulsion, Speed, Predictor, Ground reaction force

## Abstract

**Background:**

Current rehabilitation for individuals poststroke focuses on increasing walking speed because it is an indicator of community walking ability and quality of life. Propulsive force generated from the paretic limb is critical to walking speed and may reflect actual neural recovery that restores the affected neural systems. A wide variation across individuals in the improvements in paretic propulsive force was observed following an intervention that targeted paretic propulsive force. This study aimed to determine if specific baseline characteristics can be used to predict patients who would respond to the intervention.

**Methods:**

Participants (*N* = 19) with chronic poststroke hemiparesis walked at their self-selected and maximal walking speeds on a treadmill before and after a 12-week gait training program. Propulsive forces from the paretic limb were analyzed. Pearson correlation coefficient was used to determine the relationships between (1) treatment gains in walking speed and propulsive force following intervention, and (2) treatment gains in propulsive force and baseline propulsive forces.

**Results:**

Treatment gains in self-selected walking speed were correlated to treatment gains in paretic propulsive force following intervention. In addition, changes in paretic propulsive force between self-selected and maximal walking speeds at baseline were strongly correlated to treatment gains in paretic propulsive force.

**Conclusions:**

The capacity to modulate paretic propulsive force, rather than the absolute propulsive force during self-selected or maximal walking speed, predicted treatment gains in propulsive force following the intervention. Findings from this research could help to inform clinicians and researchers to target the appropriate patient population for rehabilitation interventions.

## Background

Stroke is the leading cause of long term disability in the United States [1]. Current rehabilitation for individuals poststroke focuses on increasing walking speed because it is an indicator of community walking ability and quality of life [2–5]. Increases in walking speed can be achieved via neural recovery or the development of compensation strategies [6, 7]. Specifically, during walking, propulsive forces generated from both limbs contribute to the forward progression of the body center of mass. However, because of the reduced propulsive force generated from the paretic limb in individuals poststroke [8, 9], it was observed that more severely impaired stroke patients use the propulsive force generated from the non-paretic limb to compensate [10]. Indeed, previous intervention studies have found that subjects were likely to use compensatory strategies during training, leading to increases in walking speed due to promoting compensatory strategies rather than neural recovery [9, 11–13]. Thus, evaluating treatment gains in walking speed alone may be insufficient to represent neural recovery. In contrast, the propulsive force from the paretic extremity is a direct measure of the paretic limb’s contribution and, therefore, may reflect actual neural recovery that restores the affected neural systems. Thus, recent rehabilitation research has emphasized the importance of improving paretic propulsive ability [9, 14].

Our laboratory has designed an intervention that targets paretic propulsion in individuals poststroke to increase walking speed [14, 15]. Specifically, we hypothesized that an intervention combining treadmill gait training at maximal speed and functional electrical stimulation applied to the paretic ankle musculature (FastFES) would facilitate the translation of increased plantarflexor activity into forward propulsion, ultimately resulting in increased walking speed. Although a previous preliminary study from our laboratory has reported improvements in paretic propulsive force following 12-weeks of FastFES intervention [14], we observed a wide variation across individuals in the improvements in paretic propulsive force in response to the intervention. The primary purpose of this study was to determine if specific baseline characteristic can be used to predict patients who would respond to this intervention. We first examined the relationship between gains in walking speed and gains in paretic propulsive force following the FastFES intervention. Next, baseline propulsive forces were used in a linear regression model to predict gains in propulsive force following training. We hypothesized that baseline measurement of propulsive force may reflect those individuals most likely to increase propulsive force with FastFES training. Findings from this research would better inform clinicians and researchers to target the appropriate patient population for rehabilitation.

## Methods

### Participants

A total of 19 participants (age, 60.3 ± 11.4 years; time since stroke, 6.3 ± 9.2 years; 5 female; 5 right hemiparetic; self-selected walking speed, 0.77 ± 0.32 m/s) with poststroke hemiparesis were included in this study. Participant inclusion criteria were a single cortical or subcortical stroke, a poststroke duration of at least 6 months, the ability to ambulate without the assistance of another individual, sufficient cognitive function to follow instruction and communicate with the investigators, the ability to walk for 6 min without orthotic support, sufficient passive dorsiflexion range of motion to position the ankle in a neutral position with the knee extended, and sufficient passive hip extension to extend the hip 10°. Individuals were excluded from participating if they had a history of multiple strokes, cerebellar stroke, lower extremity joint replacement, bone or joint problems that limited their ability to walk, a resting heart rate outside of the range of 40 to 100 beats per minute, a resting blood pressure outside of the range of 90/60 to 170/90 mmHg, neglect and hemianopia, unexplained dizziness during the past 6 months, or chest pain or shortness of breath without exertion. This study was approved by the Institutional Review Board of the University of Delaware and all participants provided written informed consent to participate in this study.

### Gait evaluation

Evaluations were performed at baseline (pre) and after 12 weeks of gait training (post). Kinetic and kinematic data were collected via an 8-camera motion analysis system (Motion Analysis Corp., Santa Rosa, CA, USA) as participants walked at their self-selected (SS) and fast (FS) speeds on a split-belt treadmill (Bertec Corp., Columbus, OH, USA) instrumented with 2 independent 6° of freedom force plates capturing at 1080 Hz. Previous work has described in detail the gait analysis setup [14, 16, 17]. Participants wore an overhead support harness with no body weight support and held on to a handrail (if needed) for safety. Self-selected walking speed was defined as the participant’s comfortable over ground walking speed during a 10-m walk test [18] and fast walking speed was the fastest speed that participants could maintain for at least 4 min of continuous walking on the treadmill. Kinematic and kinetic data were filtered using a bi-directional Butterworth low-pass filter at 6 and 30 Hz, respectively. Previous studies have used peak [19–23] or AGRF impulse (force-time integral) to characterize propulsion. A recent investigation from our laboratory has observed much stronger correlations between peak anterior ground reaction force (AGRF) and walking speed compared with AGRF impulse (unpublished observations). Thus, peak AGRF was used in the present study. Peak AGRF was defined as the maximum anterior ground reaction force (AGRF) normalized to body weight and was averaged across strides with 30 s trial duration. Changes during baseline speed modulation were calculated as the differences between baseline measurements at self-selected and fast walking speed. Treatment gains were calculated as the differences between posttraining and baseline measurements at self-selected walking speed.

### Training

Participants were trained at the fastest speed that they could maintain for at least 4 min. Participants completed 3 sessions a week for a total of 12 weeks. Each session consisted of six 6-min bouts of walking. Participants walked on a treadmill for bouts 1 to 5 with alternating 1-min periods with and without functional electrical stimulation. During bout 6, participants walked overground without functional electrical stimulation. Rest breaks of up to 5 min were allowed between walking bouts. More detail on the intervention may be found in previous work from our laboratory [14, 16].

## Statistical analysis

The relationship between treatment gains in self-selected walking speed and treatment gains in paretic propulsive force was analyzed by using Pearson correlation coefficient. Next, baseline measurements of propulsive force at self-selected and maximal walking speeds were used in a linear regression model to predict treatment gains in the paretic propulsive force. In addition, changes in baseline propulsive force from self-selected to fast walking speed was also used in a linear regression model to predict treatment gains in propulsive force. The significance level was set at an alpha of 0.05. All statistics were run using SPSS (version 23.0, SPSS, Inc.).

## Results

**Fig. 1:**
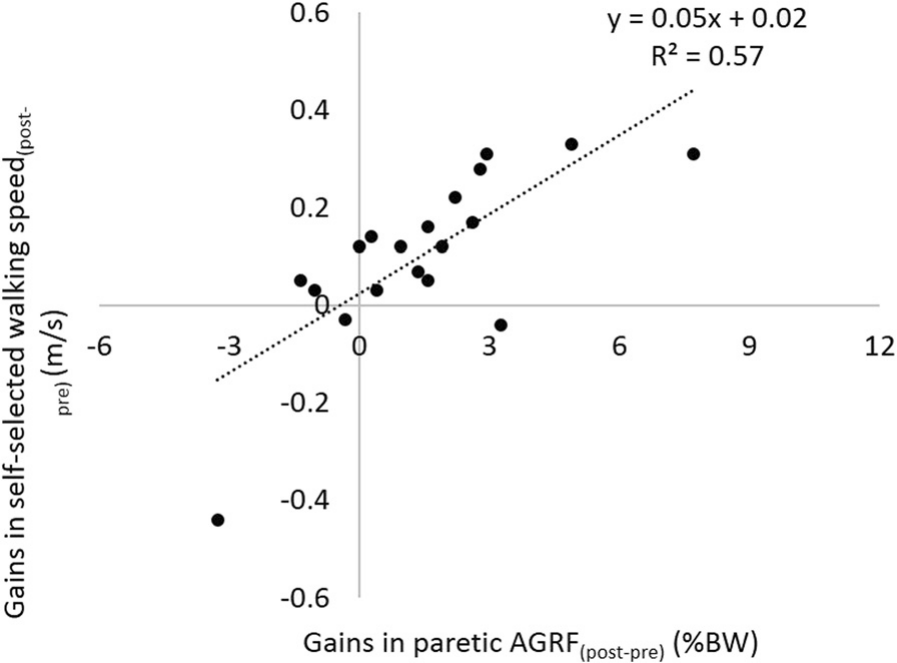
Relationship between changes in walking speed and paretic propulsive force following 12-weeks of gait training. Propulsive force was normalized to body weight. “post-pre” denotes the change from pre-training to post-training at self-selected walking speed, “BW” denotes body weight, and “*” denotes *p* < 0.01

**Fig. 2:**
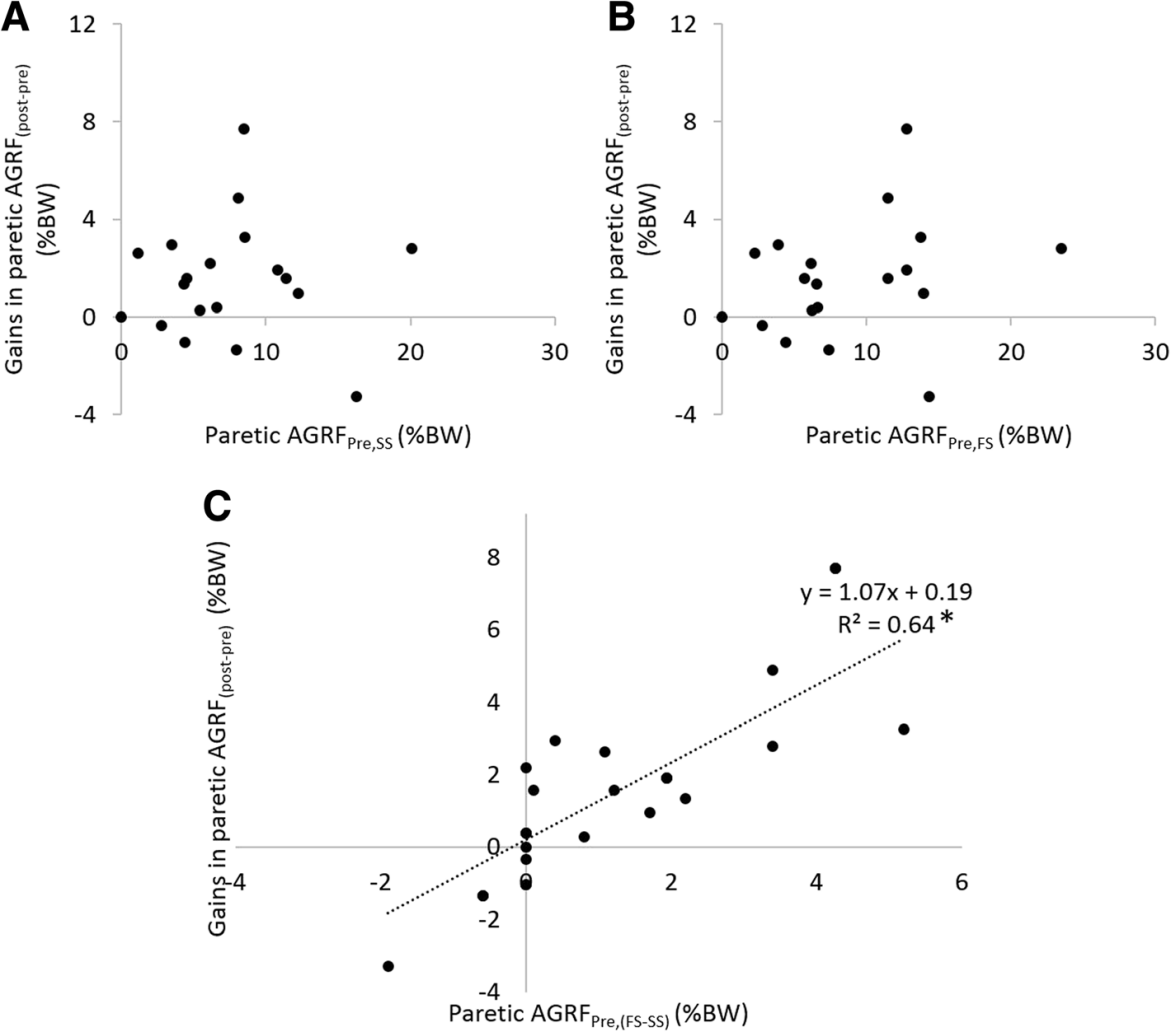
Relationships between baseline measurements and treatment gains in paretic propulsive force following intervention. **a** Baseline paretic propulsive force at self-selected walking speed versus treatment gains in paretic propulsive force. **b** Baseline paretic propulsive force at fast walking speed versus treatment gains in paretic propulsive force. **c** Changes in paretic propulsive force during baseline speed modulation versus treatment gains in paretic propulsive force. “FS-SS” denotes the change from self-selected to fast walking speed at baseline, “post-pre” denotes the change from pre-training to post-training at self-selected walking speed, “BW” denotes body weight, and “*” denotes *p* < 0.01

As expected, treatment gain in walking speed was correlated to changes in paretic propulsive force following intervention (Fig. 1, *p* < 0.01). Surprisingly, baseline paretic propulsive force at self-selected walking speed and fast walking speed were not correlated to treatment gains in paretic propulsive force (Fig. 2a-b). However, a strong correlation was observed between changes in paretic propulsive force during baseline speed modulation and treatment gains in paretic propulsive force (Fig. 2c, *p* < 0.01).

## Discussion

In the present study, our regression model showed that baseline changes in paretic propulsive force during speed modulation before training were predictive of treatment gains in paretic propulsive force (R^2^ = 0.64). This finding suggests that evaluating individuals’ biomechanical changes to increase walking speed at baseline could help to identify the best candidates for the FastFES intervention.

Participants who had greater gains in paretic propulsive force also showed greater gains in walking speed following training (Fig. 1). This relationship indicates that neural recovery of the paretic limb contributed to the gains in walking speed following training. The observed increases in walking speed and propulsion following intervention were also reported by Combs and colleagues who studied the effects of body-weight supported treadmill training in persons with chronic stroke [11]. These results highlight the importance and feasibility of enhancing paretic propulsive force. Treatment gains in paretic propulsive force ranged from −3.3 to +7.7 %BW, demonstrating a wide range of responses to the FastFES intervention. Note that negative gains indicate decreases in propulsive force.

Our results suggest that individuals who showed the ability to modulate paretic propulsive force were more likely to benefit from the FastFES intervention (Fig. 2c). Interestingly, treatment gains in paretic propulsive were not greater either in more impaired or less impaired participants (Fig. 2a, b). In fact, the 2 individuals who gained the most paretic propulsive force showed moderate paretic propulsive forces at their self-selected and maximal walking speeds at baseline. Previous studies of baseline predictors of treatment gains have shown contradictory results [24–29]. A study in upper extremity rehabilitation in individuals with chronic stroke found that moderate to severely impaired individuals could benefit significantly after constraint-induced movement therapy [28]. In the lower extremities, it was found that in the first six months after stroke, individuals with poorer paretic lower extremity motor function at baseline had faster rates of recovery than those with greater lower extremity motor scale scores [29]. However, in the chronic stroke population, Mulroy and colleagues found that participants who improved gait velocity had greater selective motor control at baseline [24]. In interpreting the above results, it was suggested that high functioning subjects could exhibit a “ceiling effect” and may be less likely to improve. On the other hand, it is also possible that low functioning subjects may be too severely impaired and therefore would have limited ability to improve. Because both explanations pointed towards the capacity to change, we used an approach that directly assessed the ability to modulate propulsive force at baseline. The present study showed that measuring baseline capacity provided an estimate of individual treatment gains. That is, these data suggested that those individuals most likely to benefit from the intervention also demonstrated the ability to increase their propulsive forces at baseline, regardless of their absolute values.

An interesting observation was that the slope of the trendline between baseline modulation and treatment gains (see Fig. 2c) is close to 1, indicating that individuals’ paretic propulsive forces at baseline maximal speed became their propulsive forces at comfortable speed after training. It should be noted that during evaluations, participants were asked to walk at their comfortable and maximal walking speeds, no instructions were given in terms of propulsive force. Thus, individuals who showed an increase in paretic propulsive force not only have the capacity to increase it, but also voluntarily used their paretic propulsive force to increase their walking speeds. In contrast, the 2 subjects that voluntarily decreased their paretic propulsive at their maximal speed also decreased their paretic propulsive forces following training. Thus, in addition to the capacity, the mechanism that individuals used to increase walking speed at baseline could also affect treatment gains in propulsive force.

A potential limitation of this study is that we only focused on biomechanical variables. Another measurement that is clinically important is walking speed. However, we did not find relationships between baseline changes in walking speed and treatment gains in walking speed. A previous study found that functional magnetic resonance imaging measurements could predict gains in walking speed in individuals 1–12 months poststroke [30]. Thus, including measurements that assess neurological pathway integrity may have the potential to further enhance the prediction of treatment gains in walking speed. In addition, although the presented predictor was able to explain 64 % of the variances in treatment gains in propulsive force, there are other factors that could affect treatment gains in propulsive force. For example, gains in propulsive force during self-selected walking speed could be influenced by fear of falling. Future investigation using balance measurements could provide useful information. However, the current study provides alternative biomechanical measurements that could be powerful indicators of treatment gains following intervention.

## Conclusions

This is the first study that showed that the capacity to modulate paretic propulsive force, rather than the absolute propulsive force during self-selected or maximal walking speed, predicted treatment gains in propulsive force following the FastFES intervention. Findings from this research could help to inform clinicians and researchers to target the appropriate patient population for rehabilitation interventions.

## Abbreviations

AGRF: Anterior ground reaction force
SS: Self-selected
FS: Fast
